# Exposure of A2E to blue light promotes ferroptosis in the retinal pigment epithelium

**DOI:** 10.1186/s11658-025-00700-2

**Published:** 2025-02-21

**Authors:** Bo Yang, Kunhuan Yang, Yuling Chen, Qingjian Li, Jingmeng Chen, Shiying Li, Yalin Wu

**Affiliations:** 1 Department of Ophthalmology, The First Affiliated Hospital of Xiamen University, School of Medicine, Xiamen University, Xiamen, 361003 Fujian China; 2 Fujian Provincial Key Laboratory of Ophthalmology and Visual Science, Fujian Engineering and Research Center of Eye Regenerative Medicine, Eye Institute of Xiamen University, School of Medicine, Xiamen University, Xiamen, 361102 Fujian China; 3 School of Medicine, Xiamen University, Xiamen, 361102 Fujian China; 4 Shenzhen Research Institute of Xiamen University, Shenzhen, 518057 Guangdong China

**Keywords:** Lipofuscin, A2E, Ferroptosis, Blue light, Macular degeneration, Retinal pigment epithelium

## Abstract

**Background:**

Age-dependent accumulation of lipofuscin in the retinal pigment epithelium (RPE) is closely related to the etiology of autosomal recessive Stargardt’s disease (STGD1) and dry age-related macular degeneration (AMD). *N*-retinylidene-*N*-retinylethanolamine (A2E) is a leading component of RPE lipofuscin that is highly susceptible to blue light. Ferroptosis is an iron-dependent form of non-apoptotic cell death characterized by the accumulation of lipid peroxides to a lethal level, which plays an important role in retinal diseases. However, it remains unknown whether A2E functions as a physiological trigger for eliciting blue light-induced ferroptosis of RPE cells.

**Methods:**

A2E-loaded RPE cells and *Abca4*^−/−^*Rdh8*^−/−^ mice were exposed to blue light, respectively. Western blotting, immunofluorescence staining, reactive oxygen species (ROS) staining, intracellular iron staining, lipid peroxidation staining, fundus imaging, optical coherence tomography (OCT), hematoxylin–eosin (HE) staining, and electroretinography (ERG) were utilized to elucidate the role of blue light in A2E induced ferroptosis in the RPE and its potential mechanisms.

**Results:**

Exposure of A2E to blue light promoted ferroptotic cell death in RPE cells by elevating ferrous ion (Fe^2+^) levels and inhibiting the solute carrier family 7 membrane 11 (SLC7A11)-glutathione (GSH)-glutathione peroxidase 4 (GPX4) axis. GPX4 inactivation and ROS generated by Fe^2+^ overload and GSH depletion precipitated lipid peroxidation and subsequent ferroptosis in A2E-containing RPE cells upon exposure to blue light. In addition to GSH supplement, repressing either Fe^2+^ by deferiprone (DFP) or lipid peroxidation with ferrostatin-1 (Fer-1) significantly protected RPE cells against ferroptosis caused by blue light illumination of A2E. *Abca4*^−/−^*Rdh8*^−/−^ mice featured by an accelerated deposition of A2E in the RPE is an animal model for STGD1 and dry AMD. It was observed that ferroptosis was indeed present in the RPE of *Abca4*^−/−^*Rdh8*^−/−^ mice following exposure to blue light. Notably, alleviating ferroptosis by intraperitoneally injected Fer-1 effectively rescued retinal function and ameliorated RPE/photoreceptor degeneration in blue light-exposed *Abca4*^−/−^*Rdh8*^−/−^ mice.

**Conclusions:**

Our results suggest the importance of blue light in A2E-mediated ferroptosis in the RPE, and deeply broaden the understanding of mechanisms underlying RPE atrophy arising from lipofuscin accumulation in STGD1 and dry AMD.

**Supplementary Information:**

The online version contains supplementary material available at 10.1186/s11658-025-00700-2.

## Introduction

Condensation products of all-*trans*-retinal accumulate with age as lipofuscin in retinal pigment epithelium (RPE) cells of the eye and are closely associated with the pathology of autosomal recessive Stargardt’s disease (STGD1) and dry age-related macular degeneration (AMD) [1–5]. STGD1 is a juvenile macular degeneration caused by mutations in the retina-specific *ATP-binding cassette (ABC) transporter 4* (*Abca4*) gene [6]. There is already evidence suggesting that the gene *Abca4* serves as a contributing risk factor for dry AMD [7–9]. Moreover, previous reports indicate that the photoreceptor-specific all-*trans*-retinol dehydrogenase 8 (*Rdh8*) is an assistant susceptibility gene for STGD1 and dry AMD, and its knockout accelerates retinal atrophy in *Abca4* mutant mice [10, 11]. Mice with a knockout of *Abca4* and *Rdh8* genes (*Abca4*^−/−^*Rdh8*^−/−^ mice) reproduce primary features of STGD1 and dry AMD, including the deterioration of photoreceptors and the RPE as well as the accumulation of all-*trans*-retinal condensation products [11]. *N*-retinylidene-*N*-retinylethanolamine (A2E) is a product of all-*trans*-retinal condensation reactions, and it is incriminated as a major component of RPE lipofuscin [12]. Compared with wild-type mice, *Abca4*^−/−^*Rdh8*^−/−^ mice exhibit a much greater accumulation of A2E in the eye with age [11, 13]. Several lines of investigation show that A2E that accumulates beyond a critical level in RPE cells mediates the detergent-like effect on cell membranes, alkalinization of lysosomes, and detachment of proapoptotic proteins from mitochondria, as well as the induction of autophagy [4, 14–17]. A2E is a compound that is highly sensitive to 430 nm blue light [18, 19]. Upon irradiation with blue light, A2E elicits the death of RPE cells via triggering DNA damage, oxidative stress, and complement activation [20–22]. Marie and colleagues have posited that exposure to blue light at a wavelength of 430 nm is implicated in the highest levels of reactive oxygen species (ROS) and mitochondrial dysfunction in A2E-loaded RPE cells [23].

Ferroptosis is an iron-dependent form of non-apoptotic cell death characterized by the accumulation of lipid peroxides to a lethal level [24]. It is morphologically, functionally, and biochemically distinct from other forms of cell death, such as apoptosis, necrosis, and autophagy [25]. Although A2E has been shown to mediate blue light-induced apoptosis and necroptosis in RPE cells [26, 27], it remains unknown whether A2E serves as a physiological trigger for eliciting blue light-induced ferroptosis of RPE cells. In this study, we explicated the role of ferroptosis arising from blue light illumination of A2E in retinal degeneration leading to STGD1 and dry AMD.

## Methods and materials

### Reagents

A2E was synthesized as previously reported [28]. A list of reagents was provided in the Supplementary Materials Table S1.

### Animals

C57BL/6 J mice were obtained from the Xiamen University Laboratory Animal Center. *Abca4*^−/−^*Rdh8*^−/−^ mice were generated and genotyped as we previously described [29]. C57BL/6 J and *Abca4*^−/−^*Rdh8*^−/−^ mice (3 months old) were exposed to 3250-lx blue light (430 nm, 50 mW/cm^2^) from a light emitting diode (LED)-based system for 30 min following pupillary dilation with 1% tropicamide, after which they were kept in darkness for 5 days. Control groups of C57BL/6 J and *Abca4*^−/−^*Rdh8*^−/−^ mice were maintained in darkness for the same period without blue light exposure. In a treatment schedule, 3-month-old *Abca4*^−/−^*Rdh8*^−/−^ mice received intraperitoneal injections of Fer-1 (4 mg/kg) or vehicle (dimethyl sulfoxide, DMSO). A total of 1 h later, they were illuminated by 3250-lx blue light for 30 min, followed by daily treatment of DMSO or Fer-1 for an additional 4 days. Control *Abca4*^−/−^*Rdh8*^−/−^ mice received intraperitoneal injections of Fer-1 or DMSO alone without light exposure. On day 5 after blue light exposure, eyeballs were harvested and then dissected to obtain RPE/choroids or posterior eyecups containing RPE/choroids. Note that the vehicle, DMSO, was composed of 10% DMSO, 40% PEG300, 5% Tween-80, and 45% saline. The volume for each intraperitoneal injection was 200 μl.

### Cell culture and treatment

Human RPE cell line ARPE-19 (CRL-2302, ATCC) was purchased from FuDan IBS Cell Center (Shanghai, China). Culture conditions of ARPE-19 cells have been described in our previous studies [30]. ARPE-19 cells were used between passages 3 and 6 to ensure consistent results. Cells were cultured in Dulbecco’s modified eagle medium (DMEM)/F12 medium supplemented with 10% fetal bovine serum (FBS) and 1% penicillin–streptomycin, maintained in a humidified atmosphere of 5% CO_2_ at 37 °C, and used in an undifferentiated state. Monolayer-confluent ARPE-19 cells were incubated with A2E (25 and 50 μM) for 48 h, and then completely washed with PBS, ensuring that only intracellular A2E remained. Following the addition of fresh DMEM/F12 medium, the cells were illuminated for 30 min by 10,000-lx 430 nm blue light (50 mW/cm^2^) from a LED-based system and then incubated for additional 24 h. Alternatively, 25 μM A2E was incubated with monolayer-confluent ARPE-19 cells for 48 h. After completely washing the cells by PBS, the cells were pretreated with GSH (2, 4, and 6 mM) for 1 h, DFP (50, 100 and 200 μM) for 2 h or Fer-1 (20, 30, and 40 μM) for 2 h, and then exposed for 30 min to blue light, followed by 24 h of incubation.

### Cell viability

Cell viability was assessed by an MTS assay kit [31]. According to the protocol of the MTS assay kit, the treated cells were incubated with 120 μl of the detection solution for 1 h, and then the absorbance at 490 nm was measured by a microplate reader. Cell viability was obtained by comparing the absorbance of each group.

### Detection of lactate dehydrogenase (LDH) release

The LDH release levels were detected by a LDH release assay kit [31]. Following the kit’s protocol, treated cells in a 96-well plate were incubated with 10 µl LDH release reagent at room temperature in the dark for 15 min. The plate was then centrifuged at 400*g* for 5 min at room temperature, and 100 µl supernatant from each well was transferred to a new plate and mixed with 100 µl LDH detection working solution. The mixture was incubated at room temperature in the dark for 30 min. To terminate the reaction, 10 µl of stop solution was added, and the absorbance was measured at 450 nm. The relative LDH release was calculated on the basis of the absorbance values for each group.

### Detection of glutathione (GSH) levels

Intracellular GSH levels were detected using a GSH assay kit. Following the kit’s protocol, treated cells were centrifuged, and the supernatant was removed. Subsequently, 20 µl protein removal reagent was added, and the samples were thoroughly vortexed. The samples underwent two rapid freeze–thaw cycles using liquid nitrogen and a 37 °C water bath, followed by incubation at 4 °C for 5 min. The samples were then centrifuged at 10,000*g* for 10 min at 4 °C, and the supernatant was collected for glutathione analysis. The glutathione content in each group was quantified using the kit’s standard curve.

### Fluorescence imaging of ROS, intracellular iron, and lipid peroxidation

Fluorescence imaging of ROS, intracellular iron, and lipid peroxidation was conducted as previously reported [32, 33]. Cells were incubated with 10 μM Hochest 33342 and 10 μM H2DCFDA, 5 μM FerroOrange or 10 μM C11-BODIPY 581/591 at 37 °C for 30 min, and then washed with PBS for fluorescence imaging. Fluorescence images of ROS production were taken under a Leica DMi8 fluorescence microscope. Fluorescence images of intracellular iron and lipid peroxidation were photographed by a Zeiss LSM 880 confocal microscope. Fluorescence intensity was quantified by ImageJ software and shown as fold changes relative to DMSO-treated controls.

### Quantitative real-time polymerase chain reaction (qRT-PCR)

The method of qRT-PCR was executed as described previously [34]. Total RNA was extracted from cells using the total RNA extraction reagent. Subsequently, total RNA was reverse-transcribed into cDNA using the qPCR RT Master Mix kit. qRT-PCR was performed using the DNA Green Master on a Roche Light Cycler 96 system. The primer sequences used for qRT-PCR were shown in the Supplementary Materials Table S2.

### Western blotting

Cells or RPE/choroid tissues were lysed on ice for 30 min in RIPA buffer supplemented with 2% protease and phosphatase inhibitors. The lysates were centrifuged at 10,000 rpm for 10 min at 4 °C, and the protein concentration of the supernatants was determined using a BCA assay. Equal amounts of protein were resolved by sodium dodecyl-sulfate polyacrylamide gel electrophoresis (SDS-PAGE) on a 12% polyacrylamide gel, transferred onto polyvinylidene difluoride (PVDF) membranes, and blocked with 5% skim milk for 1 h at room temperature. The membranes were then incubated overnight at 4 °C with primary antibodies (1:1000 dilution), followed by three washes with TBST. Secondary antibodies (1:2000 dilution) were applied for 2 h at room temperature. Protein bands were detected using a Bio-Rad scanner. The information of antibodies used for western blotting is presented in the Supplementary Materials Table S1. Protein levels were quantified by ImageJ software and presented as fold changes relative to DMSO-treated controls. The unprocessed original images of western blot results are provided in the Supplementary Materials Fig. S1.

### Hematoxylin and eosin (HE) staining

Mouse eyeballs were embedded in paraffin and sectioned into 3 µm-thick slices, followed by HE staining. Images of HE staining were observed by a Leica DM2500 microscope, and the outer nuclear layer (ONL) thickness was measured at 250 µm intervals from the optic disc to the nasal and temporal regions.

### Immunofluorescence staining

The mouse eyeballs fixed with tissue fixative for 30 min were carefully separated under a microscope to obtain the posterior eyecup containing RPE/choroid, and then prepared into RPE/choroid flat mounts. The RPE/choroid flat mounts were permeabilized with 1% Triton for 1 h and blocked with 5% BSA for 1 h, and then incubated with anti-ZO-1 antibody or anti-acrolein antibody (1:100 dilution) at 4 °C overnight. After washing with PBS three times, the RPE flat sheets were incubated with a fluorescent secondary antibody (1:200 dilution) at room temperature for 2 h. Fluorescence images were obtained by the Zeiss LSM 880 confocal microscope.

### Fundus imaging and optical coherence tomography (OCT)

Mice were anesthetized with an intraperitoneal injection of 200 μl 1% pentobarbital sodium, and their pupils were dilated using 1% tropicamide. To maintain hydration, a drop of 0.2% carbomer solution was applied to the eyes. Fundus images were captured using a small animal retinal imaging system (Optoprobe; OPIMG-L, UK). OCT images were obtained with a small animal retinal OCT system (Optoprobe; OPIMG-L, UK), using rectangular volume scans to exhibit retinal thickness, with the optic nerve as a reference point. Retinal thickness was measured using ImageJ software.

### Electrophysiology (ERG)

Dark-adapted mice were anesthetized by isoflurane inhalation, and their eyes were kept moist with carbomer solution after pupil dilation. Full-field electroretinograms were recorded at the same time of day by an animal ERG system (Diagnosys LLC; Lowell, MA, USA). Scotopic electroretinograms were elicited using a single flash stimulus at an intensity of 10 cd s/m^2^. The responses to brief flashes were quantified by measuring the amplitudes of the a- and b-waves. All experimental procedures were conducted under dim red lighting conditions.

### Statistical analyses

Data were analyzed using GraphPad Prism software (Version 8.0, USA) and expressed as mean ± standard deviation (SD) from a minimum of three independent experiments. Statistical significance was assessed by Kruskal–Wallis test with the two-stage step-up method of Benjamini, Krieger, and Yekutieli.

## Results

### Blue light triggers ferroptosis in A2E-loaded ARPE-19 cells

**Fig. 1:**
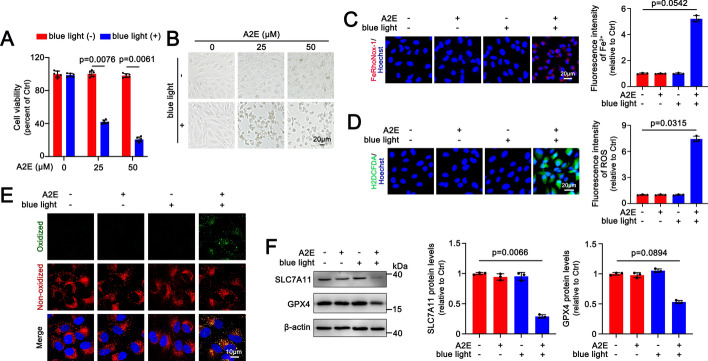
Blue light induces ferroptosis in A2E-loaded ARPE-19 cells. **A** and **B** Cell viability and cellular morphology were assessed using MTS assay and light microscopy, respectively. ARPE-19 cells were incubated with A2E (25 and 50 μM) for 48 h and then exposed to blue light for 30 min, followed by 24 h of incubation. **C**−**F** ARPE-19 cells were incubated for 48 h with 25 μM A2E and illuminated by blue light for 30 min, followed by 24 h of incubation. **C** FeRhoNox-1 staining (*n* = 3). **D** H2DCFDA staining (*n* = 3). **E** Lipid peroxidation (*n* = 3). **F** Western blot analysis of SLC7A11 and GPX4 (*n* = 3). Scale bars in **B**−**D**, 20 μm. Scale bars in **E**, 10 μm

ARPE-19 is a spontaneously arising RPE cell line derived from the normal eyes of a 19 year-old male donor [35]. ARPE-19 cells treated for 48 h with A2E at concentrations of 25 and 50 μM were completely washed by phosphate buffer saline (PBS), replenished with fresh DMEM/F12 medium, and exposed for 30 min to 10,000-lx blue light (430 nm, 50 mW/cm^2^), followed by 24 h of incubation. Cell viabilities following exposure to 25 and 50 μM A2E were not affected, but they were significantly decreased by blue light (approximately 57.99 and 79.37%, respectively) (Fig. 1A). Likewise, the morphologies of ARPE-19 cells treated with 25 and 50 μM A2E almost remained intact; however, they suffered from blue light-induced damage that was both concentration-dependent and severe (Fig. 1B). On the basis of cytotoxicity data and morphological images, ARPE-19 cells treated with 25 μM A2E were exposed to blue light in subsequent experiments. Imaging of intracellular Fe^2+^ via FeRhoNox-1 fluorescent probe showed that blue light remarkably enhanced intracellular Fe^2+^ levels in A2E-loaded ARPE-19 cells (approximately fourfold higher than the control) (Fig. 1C). On examination with H2DCFDA staining, blue light visibly increased the production of ROS in A2E-loaded ARPE-19 cells (approximately sevenfold higher than the control) (Fig. 1D). Imaging with the image-iT™ lipid peroxidation kit and confocal microscopy manifested that blue light significantly aggravated lipid peroxidation within ARPE-19 cells in response to A2E (approximately 12-fold higher than the control) (Fig. 1E and Supplementary Fig. S2A). There is already evidence suggesting that blocking the cystine/glutamate antiporter system (system Xc^−^) sequentially evokes GSH depletion, glutathione peroxidase 4 (GPX4) inactivation and lipid peroxidation, ultimately resulting in ferroptotic cell death [36]. Solute carrier family 7 membrane 11 (SLC7A11) is a component of system Xc^−^, and it plays a crucial role in the synthesis of GSH [36]. The results of western blotting demonstrated that blue light elicited a significant decrease in protein levels of SLC7A11 and GPX4 in A2E-loaded ARPE-19 cells (Fig. 1F). These lines of data imply that blue light mediates A2E-induced RPE cell ferroptosis.

### GSH depletion by A2E upon exposure to blue light promotes ferroptosis in ARPE-19 cells

**Fig. 2:**
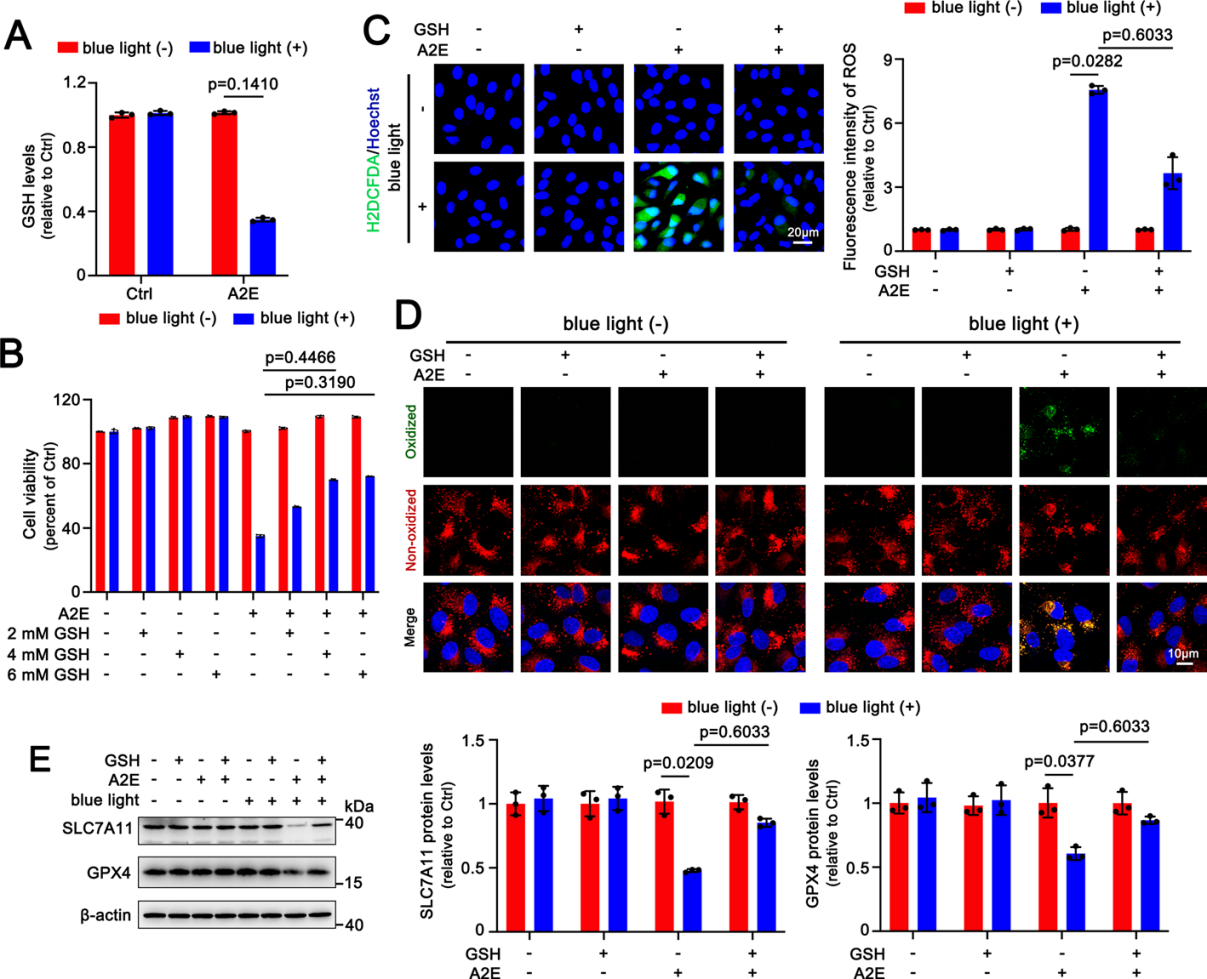
GSH depletion mediates blue light-induced ferroptosis in A2E-loaded ARPE-19 cells. **A** GSH detection (*n* = 6). ARPE-19 cells were incubated with 25 μM A2E for 48 h and then exposed to blue light for 30 min, followed by 24 h of incubation. **B** Cell viability (*n* = 6). ARPE-19 cells that were incubated with 25 μM A2E for 48 h were pretreated with serial concentrations of GSH (2, 4, and 6 mM) for 1 h and then illuminated by blue light for 30 min, followed by 24 h of incubation. **C − E** ARPE-19 cells that were treated for 48 h with 25 μM A2E were preincubated with 4 mM GSH for 1 h and exposed for 30 min to blue light, followed by 24 h of incubation. **C** H2DCFDA staining (*n* = 3). Scale bars, 20 μm. **D** Lipid peroxidation (*n* = 3). Scale bars, 10 μm. **E** Western blots of SLC7A11 and GPX4 (*n* = 3)

GSH is very important for cellular defense mechanisms against oxidative stress, and its depletion is implicated in the initiation of ferroptosis [36]. The GSH detection implied that GSH levels were significantly decreased by approximately 65% in A2E-loaded ARPE-19 cells after exposure to blue light (Fig. 2A). MTS assays revealed that GSH significantly and concentration-dependently attenuated blue light-induced toxicity of A2E in ARPE-19 cells at concentrations of 2, 4, and 6 mM, with cell viability recoveries of about 18%, 37%, and 39%, respectively (Fig. 2B). Imaging of ROS using the fluorescent probe H2DCFDA showed that 4 mM GSH significantly prevented blue light-induced ROS production in A2E-loaded ARPE-19 cells, resulting in an inhibitory rate of approximately 50% (Fig. 2C). Moreover, 4 mM GSH obviously mitigated the levels of lipid peroxidation in A2E-loaded ARPE-19 cells following exposure to blue light, leading to an inhibitory rate of approximately 77% (Fig. 2D and Fig. S2B). Western blot analysis also disclosed that 4 mM GSH distinctly increased protein levels of SLC7A11 and GPX4 in A2E-loaded ARPE-19 cells after exposure to blue light (Fig. 2E). These data suggest that GSH depletion caused by blue light irradiation of A2E facilitates RPE cell ferroptosis.

### Inhibiting Fe^2+^ alleviates ferroptosis of A2E-loaded ARPE-19 cells after exposure to blue light

**Fig. 3:**
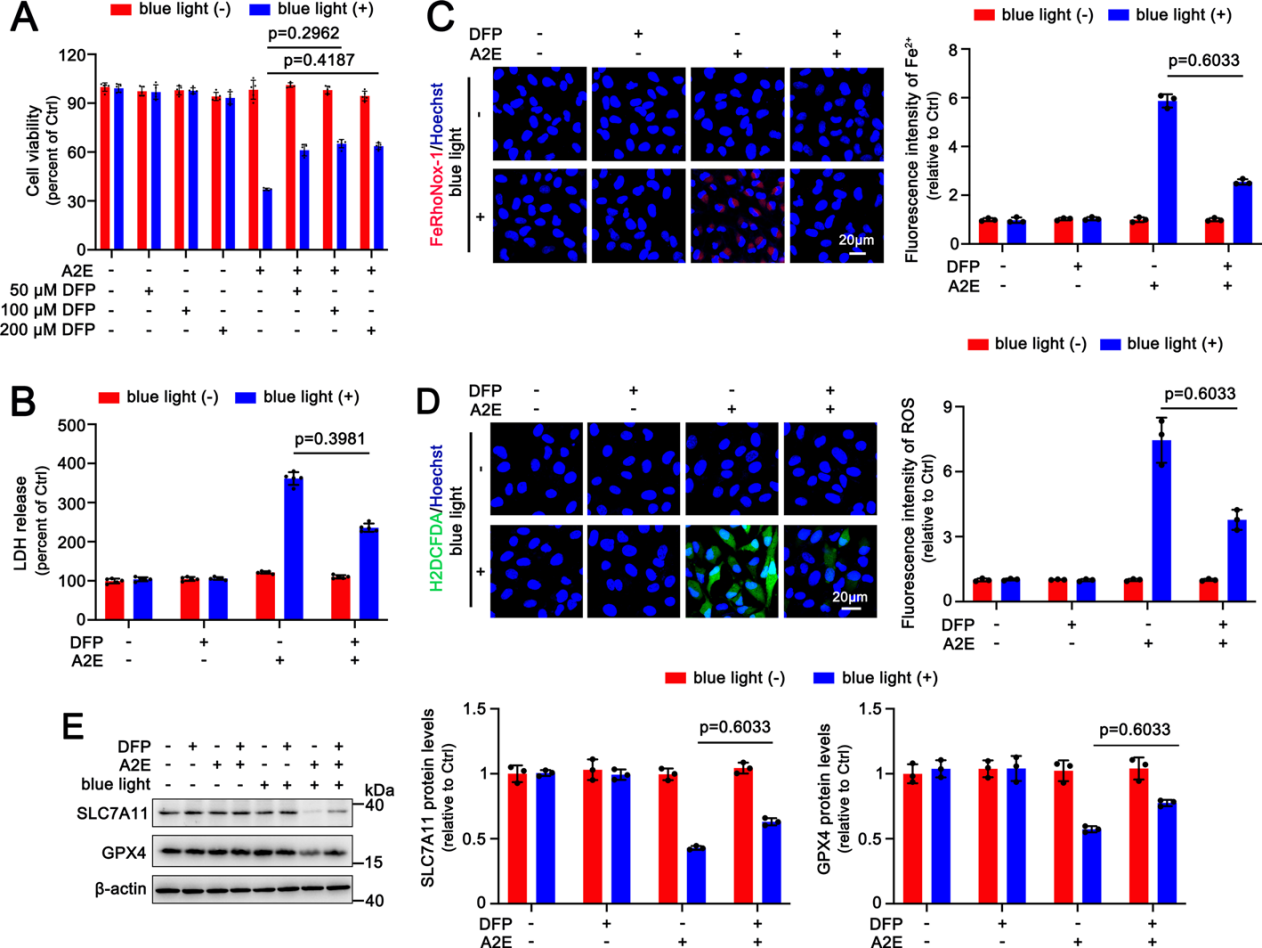
Iron-chelating agent DFP relieves blue light-induced ferroptosis in A2E-loaded ARPE-19 cells. **A** Cell viability (*n* = 6). ARPE-19 cells that were incubated with 25 μM A2E for 48 h were pretreated with serial concentrations of DFP (50, 100, and 200 μM) for 2 h and then illuminated by blue light for 30 min, followed by 24 h of incubation. **B**−**E** ARPE-19 cells that were treated for 48 h with 25 μM A2E were preincubated with 100 μM DFP for 2 h and exposed for 30 min to blue light, followed by 24 h of incubation. **B** LDH release detection (*n* = 6). **C** FeRhoNox-1 staining (*n* = 3). **D** H2DCFDA staining (*n* = 3). **E** Western blots of SLC7A11 and GPX4 (*n* = 3). Scale bars in **C** and **D**, 20 μm

Defriprone (DFP) is a well-accepted ferrous ion (Fe^2+^) chelator [37]. The MTS assay revealed that treatment with DFP at different concentrations (50, 100, and 200 μM) significantly remitted blue light-mediated toxicity in A2E-accumulating ARPE-19 cells, with recovery rates of about 20%, 32%, and 25%, respectively (Fig. 3A). Membrane integrity analysis assessed via lactate dehydrogenase (LDH) release verified that 100 μM DFP effectively inhibited LDH release from A2E-loaded ARPE-19 cells following blue light exposure, resulting in an inhibitory rate of approximately 40% (Fig. 3B). Using FeRhoNox-1 staining to detect intracellular Fe^2+^, it was found that 100 μM DFP remarkably downregulated the levels of Fe^2+^ in A2E-loaded ARPE-19 cells in response to blue light, with an inhibitory rate of about 50% (Fig. 3C). Moreover, 100 μM DFP clearly decreased the generation of ROS in A2E-loaded ARPE-19 cells with blue light illumination, leading to an inhibitory rate of approximately 43% (Fig. 3D). Analysis of western blotting also confirmed that protein levels of SLC7A11 and GPX4 were dramatically upregulated by 100 μM DFP in A2E-loaded ARPE-19 cells following exposure to blue light (Fig. 3E). These findings imply that blue light evokes ferroptotic cell death in A2E-loaded ARPE-19 cells by increasing Fe^2+^ levels.

### Ferroptosis inhibitor Fer-1 mitigates blue light-induced toxicity of A2E in ARPE-19 cells

**Fig. 4:**
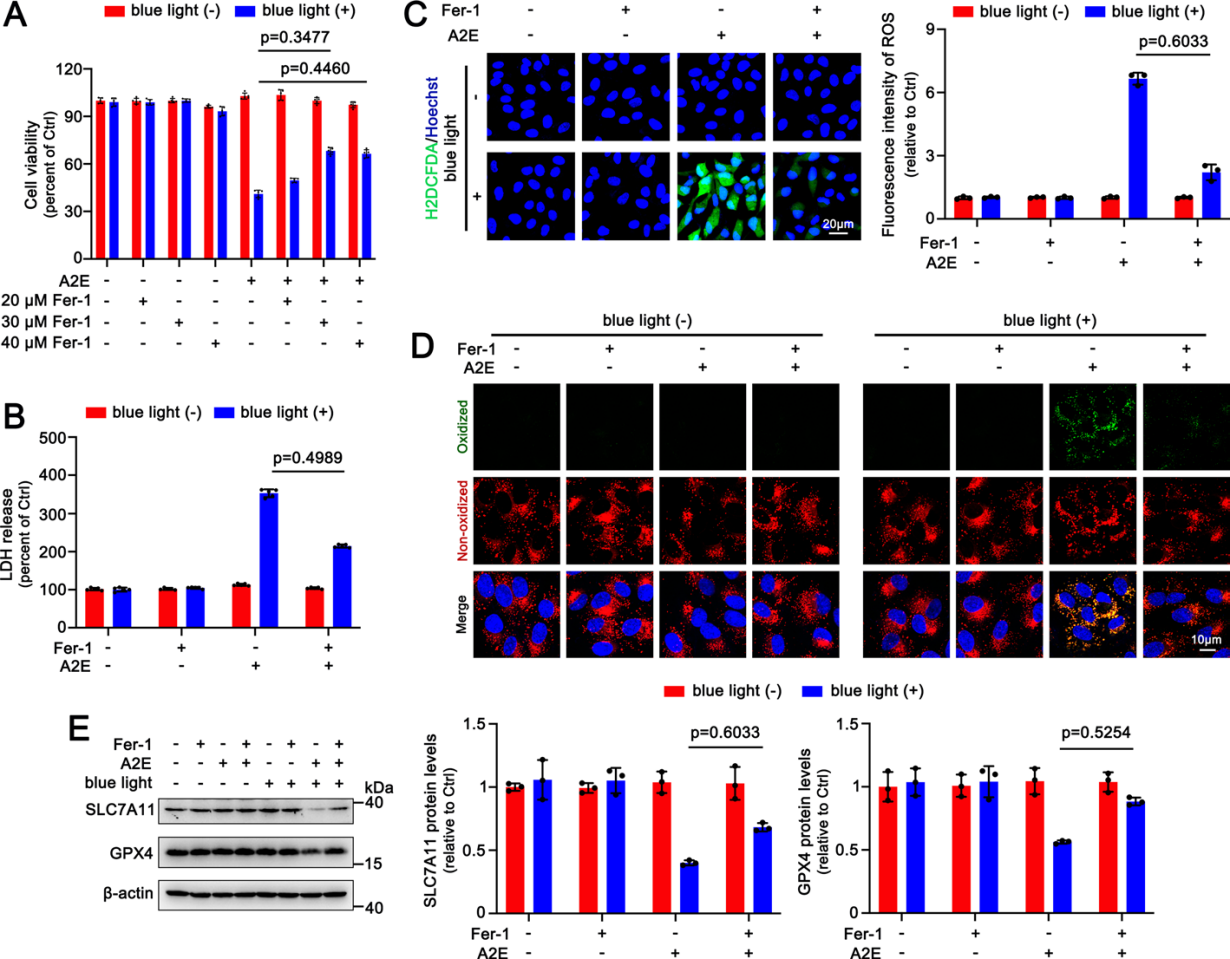
Ferroptosis inhibitor Fer-1 mitigates blue light-induced ferroptosis in A2E-loaded ARPE-19 cells. **A** Cell viability (*n* = 6). ARPE-19 cells that were incubated with 25 μM A2E for 48 h were pretreated with serial concentrations of Fer-1 (20, 30 and 40 μM) for 2 h and then illuminated by blue light for 30 min, followed by 24 h of incubation. **B**−**E** ARPE-19 cells that were treated for 48 h with 25 μM A2E were preincubated with 30 μM Fer-1 for 2 h and exposed for 30 min to blue light, followed by 24 h of incubation. **B** LDH release detection (*n* = 6). **C** H2DCFDA staining (*n* = 3). Scale bars, 20 μm. **D** Lipid peroxidation (*n* = 3). Scale bars, 10 μm. **E** Western blots of SLC7A11 and GPX4 (*n* = 3)

Ferrostatin-1 (Fer-1) is a selective ferroptosis inhibitor [38]. The MTS assay revealed that treatment with Fer-1 (20, 30, and 40 μM) remarkably restored the viability of A2E-loaded ARPE-19 cells after exposure to blue light, with recovery rates of about 6%, 25%, and 22%, respectively (Fig. 4A). As expected, 30 μM Fer-1 clearly reduced the release of LDH from A2E-loaded ARPE-19 cells following exposure to blue light, with an inhibitory rate of approximately 42% (Fig. 4B). Treatment with 30 μM Fer-1 significantly reduced the production of ROS in A2E-loaded ARPE-19 cells after exposure to blue light, achieving an inhibitory rate of approximately 64% (Fig. 4C). Moreover, 30 μM Fer-1 observably mitigated lipid peroxidation in A2E-loaded ARPE-19 cells in response to blue light, with an inhibitory rate of about 59% (Fig. 4D and Supplementary Fig. S2C). It was also found that 30 μM Fer-1 markedly prevented the downregulation of SLC7A11 and GPX4 expression in A2E-loaded ARPE-19 cells after blue light exposure (Fig. 4E). These lines of evidence suggest that repression of ferroptosis rescues RPE cells against toxicity from blue light illumination of A2E.

### Blue light-induced retinal degeneration in ***Abca4***^***−/−***^***Rdh8***^***−/−***^ mice involves ferroptosis in the RPE

**Fig. 5:**
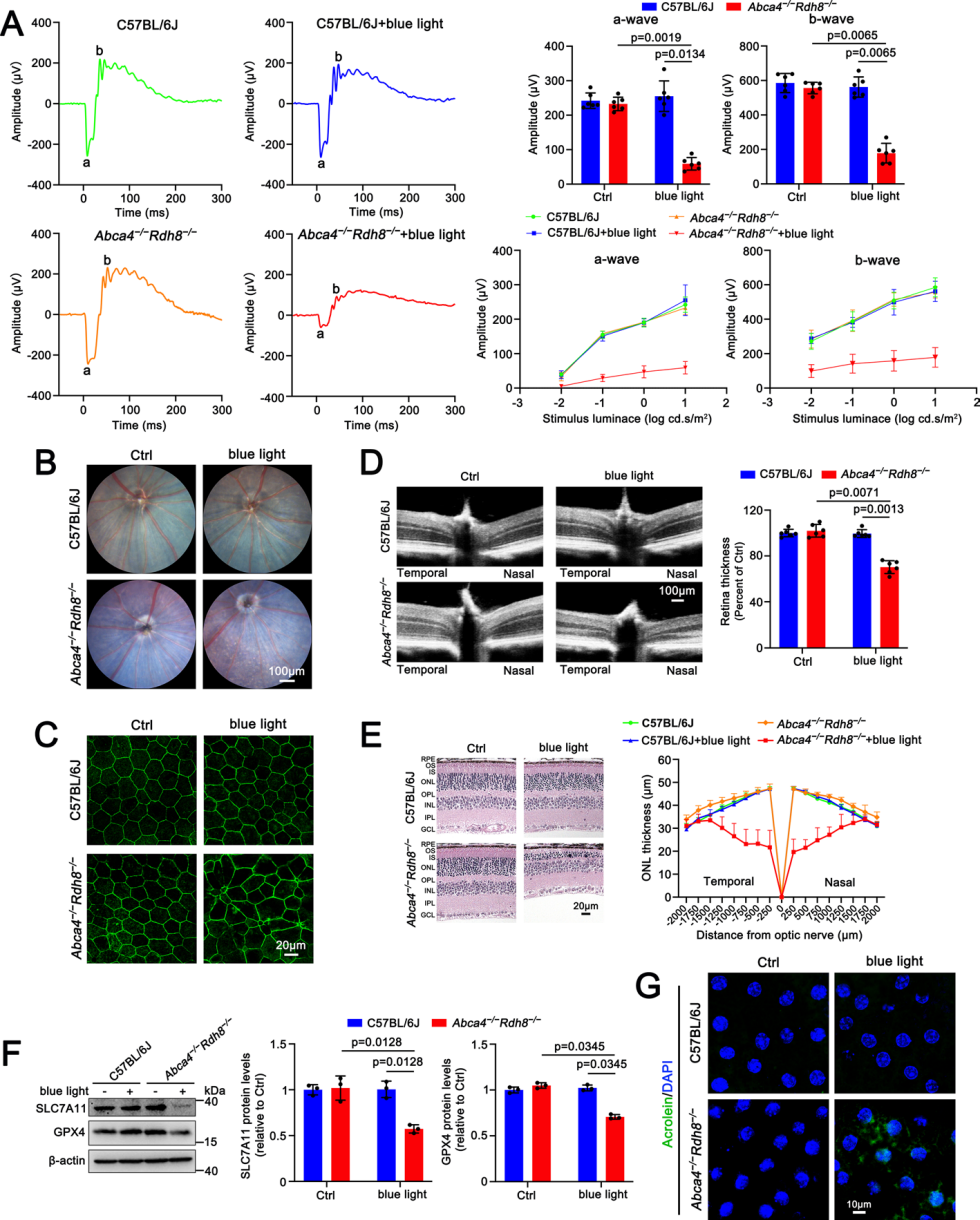
Ferroptosis involves blue light-induced retinal degeneration in *Abca4*^−/−^*Rdh8*^−/−^ mice. C57BL/6 J and *Abca4*^−/−^*Rdh8*^−/−^ mice at 3 months of age were exposed to 430 nm blue light for 30 min and then dark adapted for 5 days. Control C57BL/6 J and *Abca4*^−/−^*Rdh8*^−/−^ mice were normally raised for 5 days in the dark without exposure to blue light. **A** Full-field ERG was used to evaluate mouse retinal function under scotopic conditions. The a- and b-wave amplitudes of scotopic ERG were measured at an intensity of 10 cd s/m^2^ by using a computer-based system (*n* = 6). Alternatively, the a- and b-wave amplitudes of scotopic ERG were measured at serial intensities of 0.01, 0.1, 1, and 10 cd s/m^2^ (*n* = 6). **B** Fundus imaging. **C** ZO-1 staining of the RPE/choroid. **D** OCT imaging (*n* = 6). **E** HE staining. ONL thickness measurement on the temporal-nasal meridian (*n* = 6). **F** Western blots of SLC7A11 and GPX4 (*n* = 3). **G** Lipid peroxidation in the RPE/choroid (*n* = 3). RPE, retinal pigment epithelium; OS, outer segment; IS, inner segment; ONL, outer nuclear layer; OPL, outer plexiform layer; INL, inner nuclear layer; IPL, inner plexiform layer; GCL, ganglion cell layer. Scale bars, 20 μm

Previous studies have shown that A2E abundantly accumulates in eyes of 3-month-old *Abca4*^−/−^*Rdh8*^−/−^ mice compared with age-matched C57BL/6 J mice [13]. C57BL/6 J and *Abca4*^−/−^*Rdh8*^−/−^ mice at 3 months of age were exposed to 3250-lx blue light (430 nm, 50 mW/cm^2^) for 30 min and then housed in the dark for 5 days. Control groups of C57BL/6 J and *Abca4*^−/−^*Rdh8*^−/−^ mice were maintained under standard conditions without blue light exposure for the same duration. It should be mentioned here that dysfunction of RPE cells leads to the progression of photoreceptor degeneration [39]. The data from the evaluation of photoreceptor responses to light stimulation under scotopic condition by full-field ERG manifested that ERG amplitudes were substantially reduced in *Abca4*^−/−^*Rdh8*^−/−^ after exposure to blue light, indicating a significant loss of retinal function (Fig. 5A). The ocular fundus imaging exhibited a severe degeneration in the RPE of *Abca4*^−/−^*Rdh8*^−/−^ mice following blue light illumination (Fig. 5B). ZO-1 immunofluorescence staining confirmed that the RPE tight junctions of *Abca4*^−/−^*Rdh8*^−/−^ mice were remarkably disrupted by blue light (Fig. 5C). On examination by OCT, the thickness of neural retina was visibly reduced in *Abca4*^−/−^*Rdh8*^−/−^ mice with blue light illumination (Fig. 5D). HE staining revealed pronounced histological damage in blue light-exposed *Abca4*^−/−^*Rdh8*^−/−^ mice, characterized by a noticeable reduction in the thickness of the neural retina and the photoreceptor ONL (Fig. 5E). Conversely, blue light did not affect retinal function and the morphologies of RPE and neural retina in C57BL/6 J mice (Fig. 5A–E). Western blot analysis manifested a significant reduction in the expression of SLC7A11 and GPX4 in the RPE/choroid of *Abca4*^−/−^*Rdh8*^−/−^ mice with blue light irradiation, while their expression levels remained largely unaffected in both control *Abca4*^−/−^*Rdh8*^−/−^ mice and blue light-irradiated C57BL/6 J mice (Fig. 5F). Moreover, immunofluorescence staining for acrolein revealed a substantial increase in lipid peroxidation in the RPE/choroid of *Abca4*^−/−^*Rdh8*^−/−^ mice in response to blue light (Fig. 5G and Fig. S3A). These findings hint that ferroptosis-mediated retinal degeneration in *Abca4*^−/−^*Rdh8*^−/−^ mice is, at least in part, associated with blue light illumination of A2E in the RPE.

### Ferroptosis inhibition by Fer-1 mitigates blue light-irradiated retinal degeneration in ***Abca4***^***−/−***^***Rdh8***^***−/−***^ mice

**Fig. 6:**
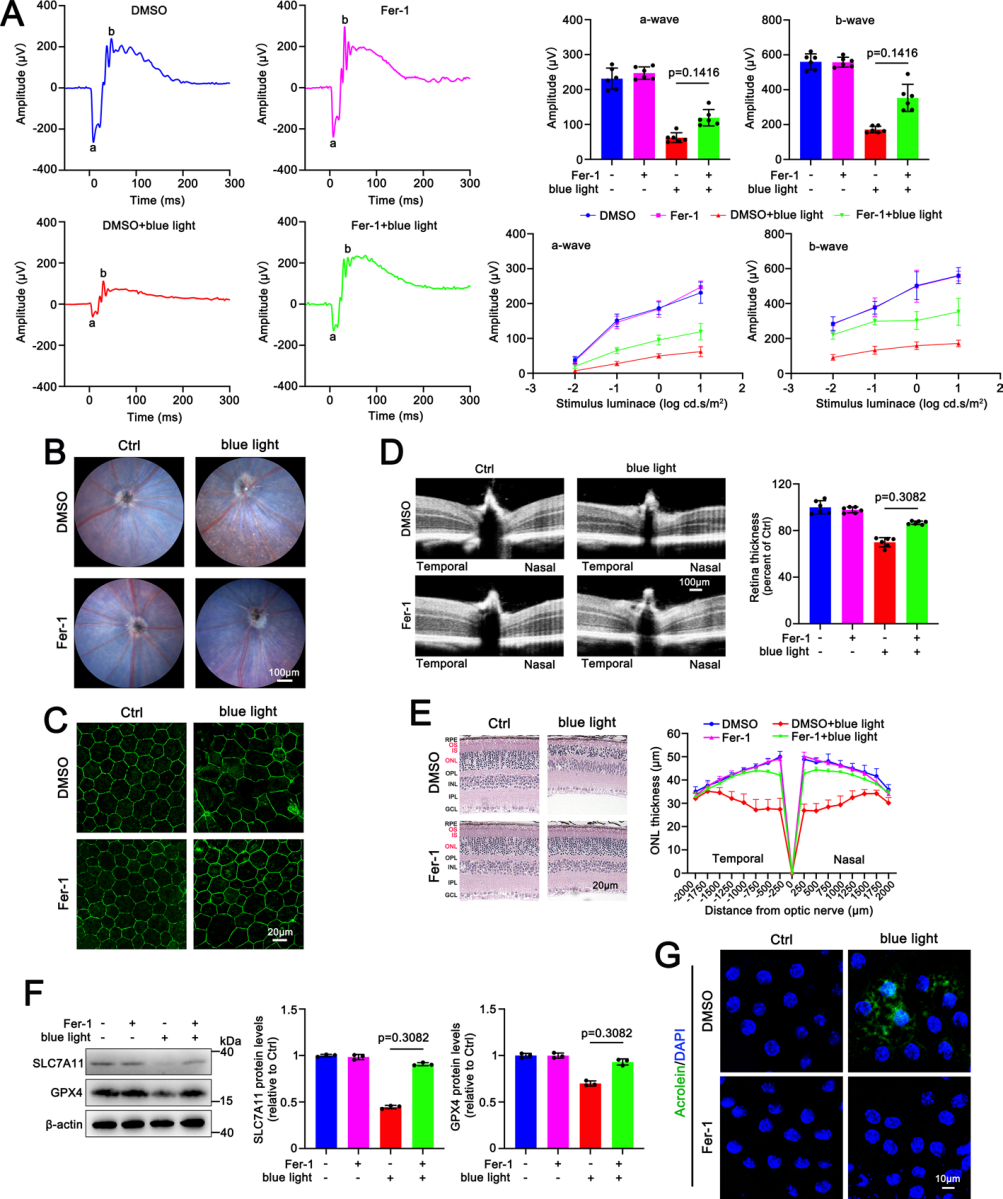
Fer-1 alleviates blue light-induced retinal degeneration and RPE cell ferroptosis in *Abca4*^−/−^*Rdh8*^−/−^ mice; 3-month-old *Abca4*^−/−^*Rdh8*^−/−^ mice were injected intraperitoneally with DMSO or Fer-1 (4 mg/kg). A total of 1 h later, the mice were irradiated by blue light for 30 min after their pupils were dilated, followed by once daily administration of DMSO or Fer-1 for additional 4 days. **A** Full-field ERG was utilized to detect retinal function under scotopic conditions. The a- and b-wave amplitudes of scotopic ERG were measured at an intensity of 10 cd s/m^2^ by using a computer-based system (*n* = 6). Alternatively, the a- and b-wave amplitudes of scotopic ERG were measured at serial intensities of 0.01, 0.1, 1, and 10 cd s/m^2^ (*n* = 6). **B** Fundus imaging.** C** ZO-1 staining of the RPE/choroid. **D** OCT imaging (*n* = 6). **E** HE staining (*n* = 6). **F** Western blots of SLC7A11 and GPX4 (*n* = 3). **G** Lipid peroxidation in the RPE/choroid (*n* = 3)

Ferroptosis repressor Fer-1 was intraperitoneally injected into blue light-exposed *Abca4*^−/−^*Rdh8*^−/−^ mice that were of the age of 3 months. The results of ERG indicated that Fer-1 effectively prevented blue light-induced drop in the amplitudes of a- and b-waves in *Abca4*^−/−^*Rdh8*^−/−^ mice, reflecting that inhibiting ferroptosis clearly improves retinal function in blue light-exposed 3-month-old *Abca4*^−/−^*Rdh8*^−/−^ mice (Fig. 6A). Retinal fundus imaging showed that RPE degeneration in *Abca4*^−/−^*Rdh8*^−/−^ mice in response to blue light was obviously attenuated by intraperitoneal administration of Fer-1 (Fig. 6B). Immunofluorescence analysis of ZO-1 revealed that treatment with Fer-1 significantly restored tight junctions in the RPE of *Abca4*^−/−^*Rdh8*^−/−^ mice upon exposure to blue light (Fig. 6C). In addition to relieving RPE degeneration, intraperitoneal injection of Fer-1 distinctly attenuated damage to neural retina and partially restored the blue light-induced decrease in the thickness of both the neural retina and the photoreceptor ONL in *Abca4*^−/−^*Rdh8*^−/−^ mice (Fig. 6D and E). As expected, western blotting manifested that Fer-1 treatment significantly enhanced protein expression of SLC7A11 and GPX4 in the RPE/choroid from *Abca4*^−/−^*Rdh8*^−/−^ mice with blue light illumination (Fig. 6F). Furthermore, immunofluorescence staining of acrolein revealed that lipid peroxidation in the RPE from blue light-irradiated *Abca4*^−/−^*Rdh8*^−/−^ mice was clearly mitigated by Fer-1 treatment (Fig. 6G and Supplementary Fig. S3B). These data suggest that ferroptosis could represent a potential therapeutic target for ameliorating retinal degeneration caused by blue light illumination of A2E within the RPE of *Abca4*^−/−^*Rdh8*^−/−^ mice.

## Discussion

There is currently a lack of effective treatments for dry AMD and STGD1. Dry AMD accounts for approximately 90% of AMD patients, and its advanced form, geographical atrophy, clinically manifests as severe vision loss or even blindness [40]. STGD1 represents a predominant form of inherited macular dystrophy in adolescents, and patients with STGD1 will lose vision when they reach adulthood [41]. The death of RPE cells is recognized as a common and pivotal factor in the pathogenesis of STGD1 and dry AMD. Indeed, apoptosis and ferroptosis of RPE cells have been documented in cell and mouse models of STGD1 and dry AMD [30, 33, 42, 43]. Moreover, ferroptotic cell death is also found to drive RPE degeneration induced by sodium iodate [44]. However, the causes of ferroptosis in the RPE of STGD1 and dry AMD patients still remain ambiguous. The accumulation of RPE lipofuscin fluorophores with age leads to the blinding degeneration characteristic of STGD1 and dry AMD [27]. A2E is a major component of lipofuscin in the RPE and its role in RPE atrophy has received the most extensive attention [45]. Blue light is a high energy visible light with a wavelength ranging from 400 to 500 nm that is largely present in sunlight and electronic products of living environments for human beings. More importantly, blue light can penetrate the lens directly to the retina where it is absorbed at least in part by A2E. There are already reports that illumination with blue light aggravates and accelerates the toxicity of A2E to RPE cells [20–22]. In this article, we demonstrated that exposure of A2E to blue light evoked RPE degeneration via activating ferroptosis.

A2E in the eye of *Abca4*^−/−^*Rdh8*^−/−^ mice accumulates with age, and its levels in the eye of 3-month-old *Abca4*^−/−^*Rdh8*^−/−^ mice are much higher than that of 6-month-old wild-type mice, and almost identical to that of 6-month-old *Abca4*^−/−^ mice, a model of STGD1 [13]. The current study indicated that *Abca4*^−/−^*Rdh8*^−/−^ mice aged 3 months did not show the loss of retinal function or the atrophy of photoreceptors and the RPE, but they dramatically suffered from blue light-mediated damage to retinal function, photoreceptors, and the RPE (Fig. 5A–E). These results suggest that blue light may act as a promotor for the induction of RPE degeneration by A2E in *Abca4*^−/−^*Rdh8*^−/−^ mice. It is well-known that an outcome of RPE cell death is the loss of photoreceptors [39]. Indeed, the rescue of RPE degeneration by repressing ferroptosis caused by blue light illumination of A2E also ameliorated photoreceptor atrophy in *Abca4*^−/−^*Rdh8*^−/−^ mice (Fig. 6).

SLC7A11 is integral to cystine uptake and GSH biosynthesis [46]. GPX4 functions as a crucial selenoenzyme in the cellular defense against ferroptosis by catalyzing the reduction of lipid peroxides, and its activity depends on the levels of GSH [47]. GSH depletion inactivates GPX4, leading to the deposition of lethal lipid peroxides within cellular membranes and subsequent ferroptosis [48, 49]. Specific knockout of GPX4 in the RPE of mice results in oxidative stress-mediated retinal degeneration [50]. These past findings reveal the importance of the SLC7A11-GPX4 axis in the regulation of ferroptosis. Herein, we found that exposure to blue light clearly downregulated protein levels of SLC7A11 and GPX4 in A2E-loaded RPE cells and the RPE/choroid of *Abca4*^−/−^*Rdh8*^−/−^ mice (Figs. 1F and 5F). Blue light-induced decreases in protein expression of SLC7A11 and GPX4 were significantly prevented by inhibiting ferroptosis in A2E-loaded RPE cells and the RPE/choroid of *Abca4*^−/−^*Rdh8*^−/−^ mice (Figs. 3, 4, and 6). Although GSH depletion was visibly observed in A2E-loaded RPE cells in response to blue light (Fig. 2A), exogenous GSH supplement distinctly prevented blue light-induced downregulation of SLC7A11 and GPX4 in A2E-treated RPE cells (Fig. 2E). Moreover, repression of ferroptosis by DFP, Fer-1, or GSH alleviated blue light-mediated damage to A2E-loaded RPE cells, and intraperitoneal injection of Fer-1 effectively mitigated RPE degeneration of blue light-exposed *Abca4*^−/−^*Rdh8*^−/−^ mice. These results suggest that blue light promotes A2E-induced ferroptosis in the RPE by suppressing the SLC7A11-GSH-GPX4 axis.

Iron homeostasis is very important for the normal physiological function of cells in the human body [51], and its disruption leads to the accumulation of labile Fe^2+^ [52]. Excess Fe^2+^ directly catalyzes the formation of detrimental free radicals including ROS through Fenton reactions and thereby facilitates ferroptotic cell death [32, 53]. Previous studies have shown that AMD patients and animals with retinopathy, such as RCS rats and Rd10 mice, all exhibit increased iron levels in the retina [54–56]. In addition, iron overload is reported to promote geographic atrophy in the mouse retina [57]. Furthermore, chelation of excess iron by salicylaldehyde isonicotinoyl hydrazone or DFP protects RPE cells against cell death caused by diverse stimuli [58–60]. In the current study, we provide evidence that exposure of A2E to blue light markedly enhanced Fe^2+^ levels in RPE cells (Fig. 1C), and reduction of Fe^2+^ levels by Fe^2+^ chelator DFP significantly attenuated blue light-induced ROS production and ferroptosis in A2E-loaded RPE cells (Fig. 3). To confirm that blue light disrupts iron homeostasis in A2E-containing RPE cells, we examined mRNA levels of iron homeostasis-related genes by qRT-PCR. As expected, the data revealed that exposure of A2E to blue light clearly upregulated mRNA levels of *divalent metal transporter 1* (*Dmt1*), *ferritin light chain* (*Ftl*), *ferritin heavy chain* (*Fth*), *six-transmembrane epithelial antigen of prostate 3* (*Steap3*), *transferrin* (*TF*), and *transferrin receptor* (*TFRC*) in RPE cells (Supplementary Fig. S4). Functionally, DMT1 facilitates the transport of Fe^2+^ from endosomes into the cytoplasm, where some Fe^2+^ is stored in ferritin, including FTH and FTL. TF binds Fe^3+^ and interacts with TFRC, enabling cellular iron uptake via TFRC-mediated endocytosis. Within specialized endosomes, Fe^3+^ is reduced to Fe^2+^ by STEAP3. Dysregulation of these proteins leads to intracellular iron dyshomeostasis [32]. Taken together, these findings indicate that blue light illumination of A2E-loaded RPE cells causes iron dyshomeostasis followed by Fe^2+^ overload.

**Fig. 7:**
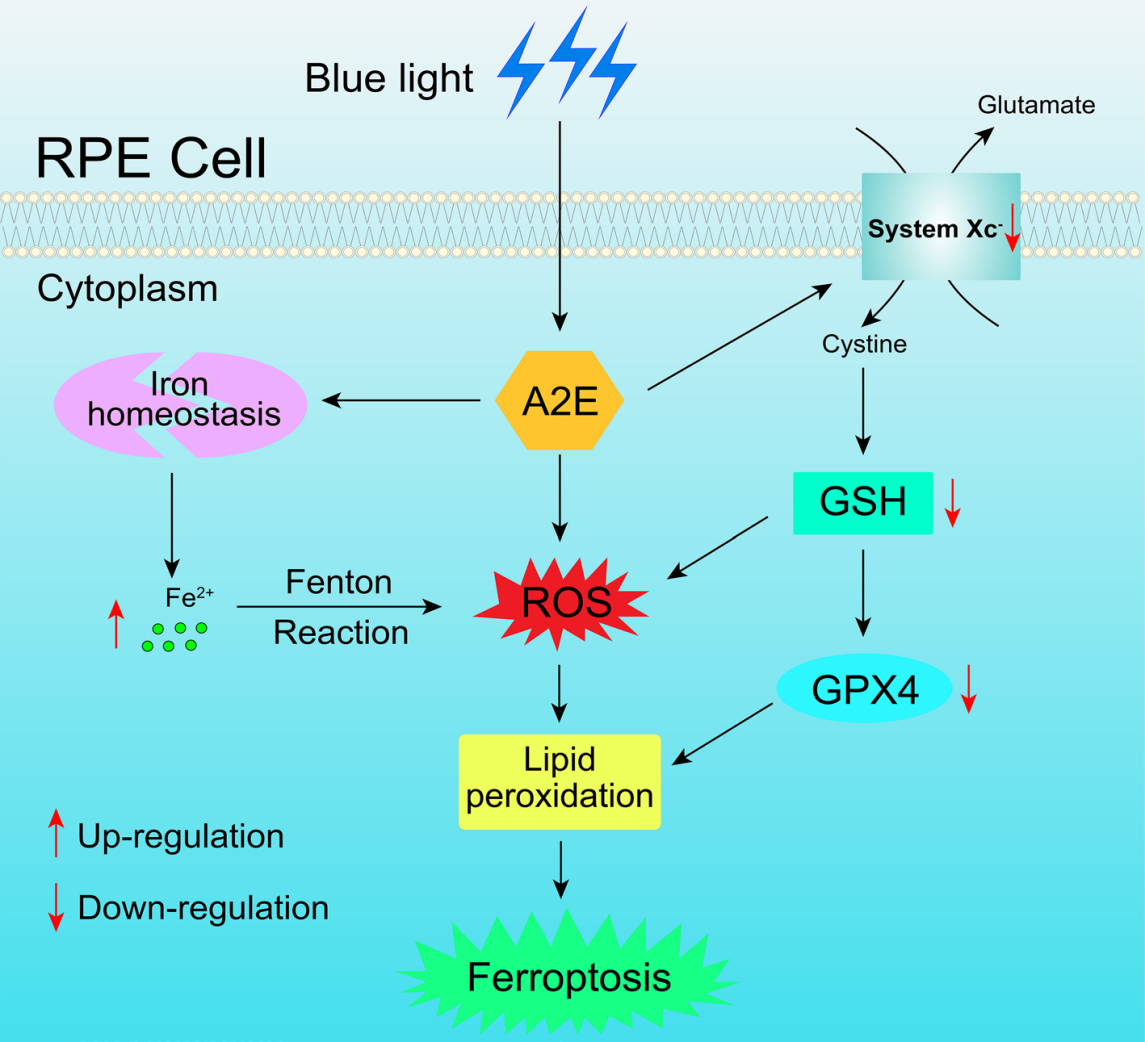
Proposed mechanisms of blue light-mediated ferroptosis in RPE cells in response to A2E. Exposure of A2E-accumulating RPE cells to blue light disrupts iron homeostasis and the function of system Xc^−^, which leads to an elevation in intracellular Fe^2+^ levels and an obvious reduction of GSH levels, respectively. In addition to generating ROS, GSH depletion inactivates GPX4. Likewise, increases in the level of Fe^2+^ cause ROS production through the Fenton reaction. GPX4 repression and ROS overproduction trigger lipid peroxidation, ultimately resulting in ferroptotic cell death

On the basis of these results, it also offers a plausible mechanism whereby blue light drives A2E-induced ferroptosis in RPE cells. As illustrated in Fig. 7, intracellular A2E that undergoes illumination by blue light disturbs system Xc^−^ to evoke a remarkably decline of GSH levels. GSH depletion can lead to GPX4 inactivation and ROS generation, thereby promoting lipid peroxidation and subsequent ferroptosis. In addition, exposure of intracellular A2E to blue light increases Fe^2+^ levels through perturbation of iron homeostasis and then instigates ROS production by Fenton reactions. Fe^2+^ overload-induced ROS incites lipid peroxidation that precipitates ferroptotic cell death. Our current work reveals the involvement of blue light in A2E-induced ferroptosis in the RPE, and expands the understanding of the underlying mechanisms of RPE degeneration resulting from the accumulation of lipofuscin in STGD1 and dry AMD.

## Supplementary Information

Supplementary Material 1. The online version contains supplementary material.

## Data Availability

The datasets used and/or analyzed during the current study are available from the corresponding author on reasonable request.

## Abbreviations

RPE: Retinal pigment epithelium
STGD1: Autosomal recessive Stargardt’s disease
AMD: Age-related macular degeneration
A2E: *N*-Retinylidene-*N*-retinylethanolamine
ROS: Reactive oxygen species
OCT: Optical coherence tomography
HE staining: Hematoxylin–eosin staining
ERG: Electroretinography
Fe^2+^: Ferrous ion
SLC7A11: Solute carrier family 7 membrane 11
GSH: Glutathione
GPX4: Glutathione peroxidase 4
DFP: Deferiprone
Fer-1: Ferrostatin-1
*Abca4*: *ATP-binding cassette (ABC) transporter 4*
*Rdh8*: All-*trans*-retinol dehydrogenase 8
LDH: Lactate dehydrogenase
*Dmt1*: *Divalent metal transporter 1*
*Ftl*: *Ferritin light chain*
*Fth*: *Ferritin heavy chain*
*Steap3*: *Six-transmembrane epithelial antigen of prostate 3*
*TF*: *Transferrin*
*TFRC*: *Transferrin receptor*
