# Supplementary material for: Exposure of A2E to blue light promotes ferroptosis in the retinal pigment epithelium
Source: Cell Mol Biol Lett. 2025 Feb 21;30:22. doi: 10.1186/s11658-025-00700-2 (PMC11846388; doi:10.1186/s11658-025-00700-2)

**Supplementary Information**

**Exposure of A2E to blue light promotes** **ferroptosis in the retinal pigment epithelium**

Bo Yang^1,2^, Kunhuan Yang^1,2^, Yuling Chen^1,2^, Qingjian Li^2^, Jingmeng Chen^3,4^, Shiying Li^1*^ and Yalin Wu^1,2,4*^

^1^ Department of Ophthalmology, the First Affiliated Hospital of Xiamen University, School of Medicine, Xiamen University, Xiamen, Fujian 361003, China

^2^ Fujian Provincial Key Laboratory of Ophthalmology and Visual Science, Fujian Engineering and Research Center of Eye Regenerative Medicine, Eye Institute of Xiamen University, School of Medicine, Xiamen University, Xiamen, Fujian 361102, China

^3^ School of Medicine, Xiamen University, Xiamen, Fujian 361102, China

^4^ Shenzhen Research Institute of Xiamen University, Shenzhen, Guangdong 518057, China

*Corresponding author. Department of Ophthalmology, the First Affiliated Hospital of Xiamen University, School of Medicine, Xiamen University, Xiamen, Fujian 361003, China.

*E-mail addresses:* [yalinw@xmu.edu.cn](mailto:yalinw@xmu.edu.cn) (Y. Wu), [shiying_li@126.com](mailto:shiying_li@126.com) (S. Li).

**Supplementary Table S1. Detailed information of reagents.**

| **Reagents** | **Catalog** | **Company** |
| --- | --- | --- |
| Deferiprone (DFP) | Y0001976 | Sigma-Aldrich |
| Ferrostatin-1 (Fer-1) | SML0583 | Sigma-Aldrich |
| Hoechst 33342 | B2261 | Sigma-Aldrich |
| 4′,6-diamidino-2-phenylindole (DAPI) | F6057 | Sigma-Aldrich |
| RIPA buffer | R0278 | Sigma-Aldrich |
| Glutathione (GSH) | G105427 | Aladdin |
| Dimethyl sulfoxide (DMSO) | D8371 | Solarbio |
| 2',7'-dichlorodihydrofluorescein diacetate (H2DCFDA) | D399 | ThermoFisher Scientific |
| Image-iT^TM^ lipid peroxidation kit | C10445 | ThermoFisher Scientific |
| Protease & Phosphatase inhibitors | 78442 | ThermoFisher Scientific |
| BCA Protein Assay Kit | 23227 | ThermoFisher Scientific |
| FeRhoNox-1 | GC901 | Goryo Chemical |
| Anti-SLC7A11 | 98051S | Cell Signaling Technology |
| Anti-β-actin | 8457S | Cell Signaling Technology |
| Anti-GPX4 | ab125066 | Abcam |
| Anti-acrolein | ab48501 | Abcam |
| Anti-ZO-1 | 33-9100 | Invitrogen |
| Alexa Fluor 488-conjugated  donkey anti-mouse ﻿secondary  antibody | A21202 | ﻿Invitrogen |
| Goat anti-rabbit IgG (H+L) secondary antibody | 31460 | Invitrogen |
| MTS assay kit | G3580 | Promega |
| LDH release assay kit | C0018 | Beyotime |
| GSH assay kit | S0053 | Beyotime |
| ﻿TRIeasy total RNA extraction reagent | D606ES60 | ﻿Yeasen |
| ﻿ReverTra Ace qPCR RT Master Mix | ESQ-201 | Toyobo |
| ﻿FastStart Essential DNA Green Master | 6402712001 | ﻿Roche |

**Supplementary Table S2. Primer sequences.**

| Gene | Forward primer | Reverse primer |
| --- | --- | --- |
| *Dmt1* | CTGTGGTCAGCGTGGCTTAT | TCAGCAGGCCTTTAGAGATGC |
| *Ftl* | TGGGCTTCTATTTCGACCGC | TCCAAAAGGGCCTGGTTCAG |
| *Fth* | CAACGAGGTGGCCGAATCTT | AAGTCACACAAATGGGGGTC |
| *Steap3* | CCCTATGTGCAGGAAAGCCA | GGGCAAGTACACGAGTGACA |
| *Tf* | TGGGAGCCCTGCTGGT | TATGGTCGCGGAAACTCTGG |
| *Tfrc* | TCGGAGAAACTGGACAGCAC | ATCACGCCAGACTTTGCTGA |

**Supplementary Figure S1. Unprocessed original western blots. Boxes in *red* indicate selected western blot results.**

**Figure 1F**


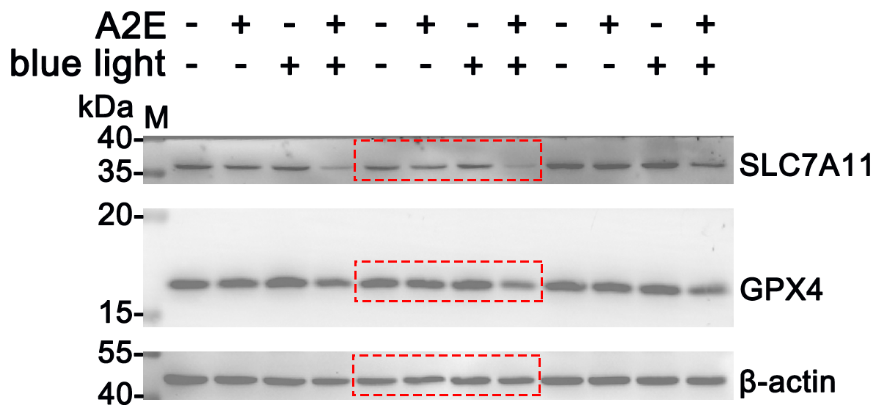


**Figure 2E**


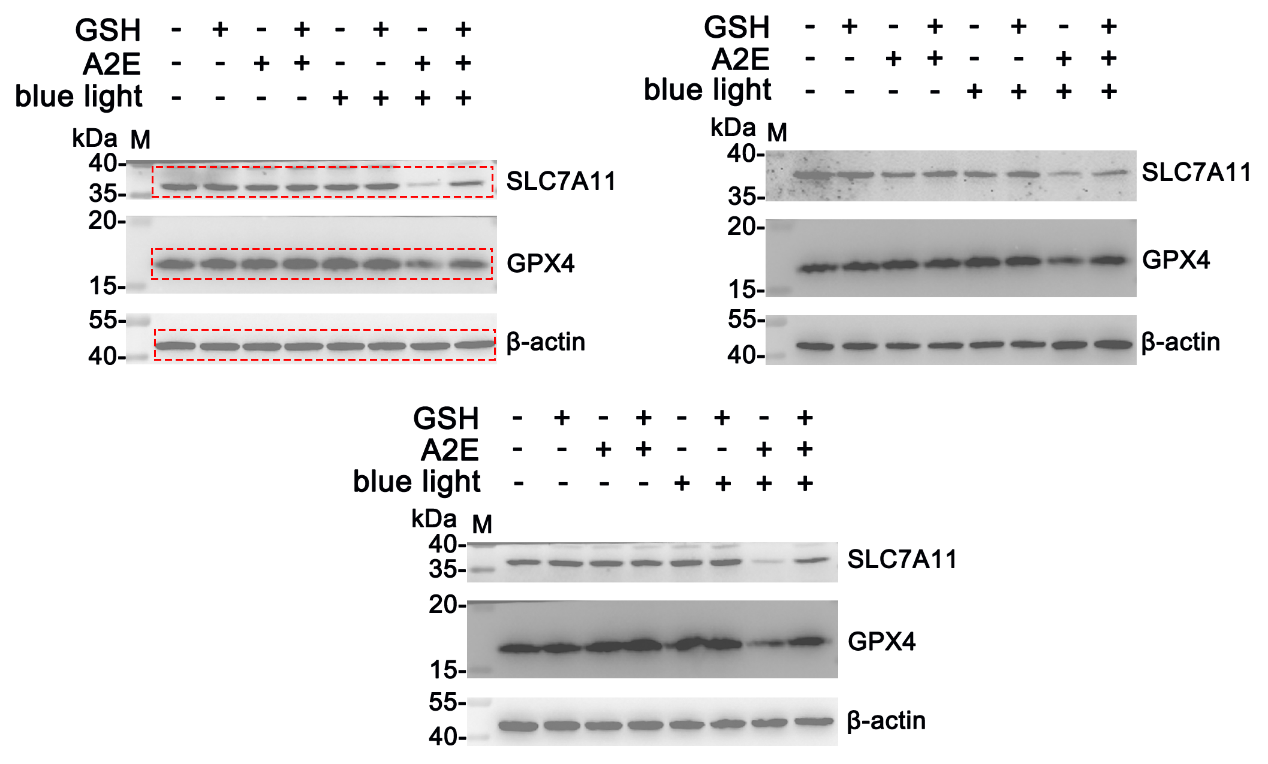


**Figure 3E**


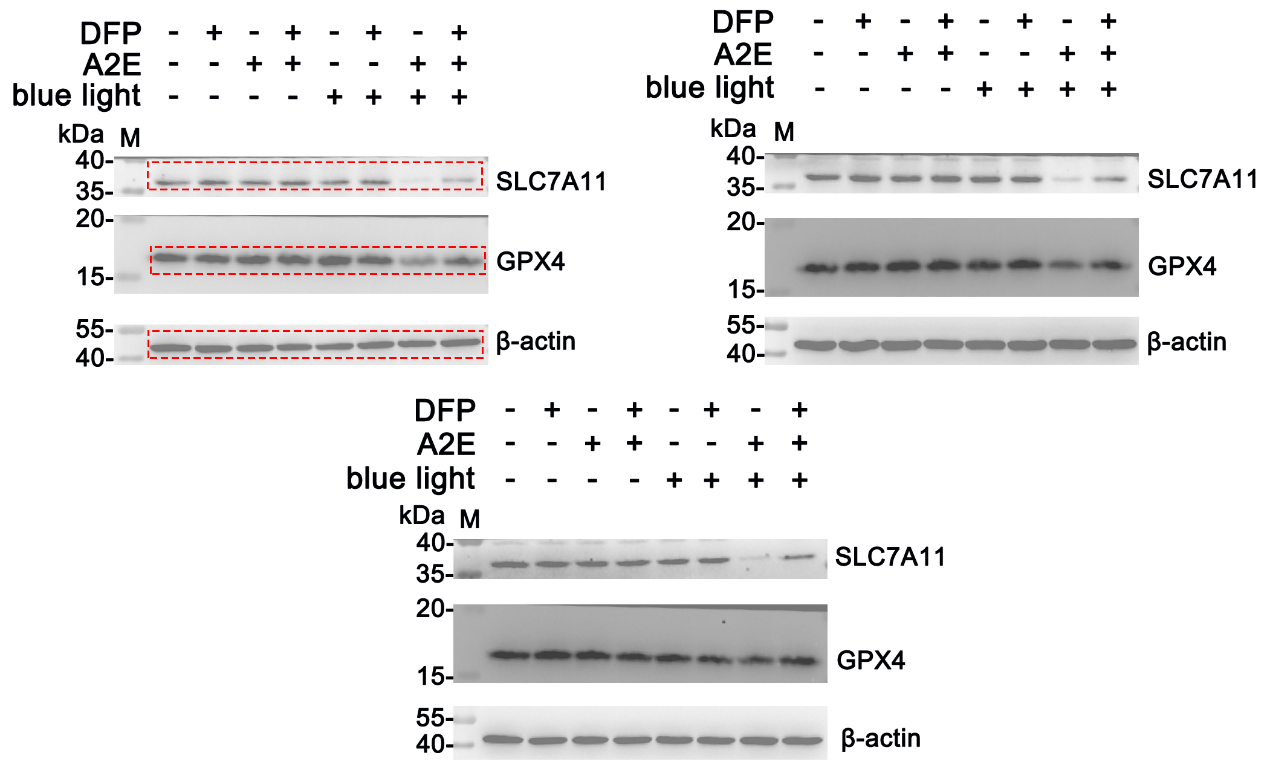


**Figure 4E**


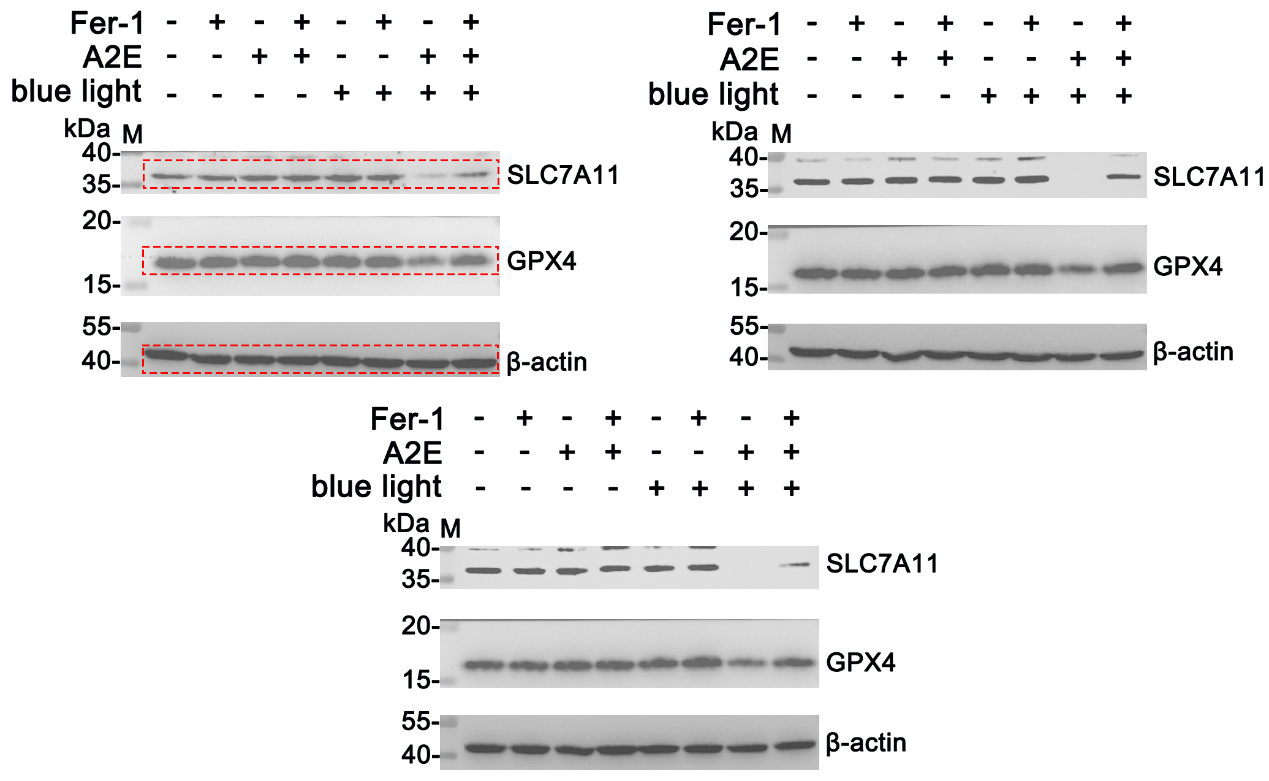


**Figure 5F**


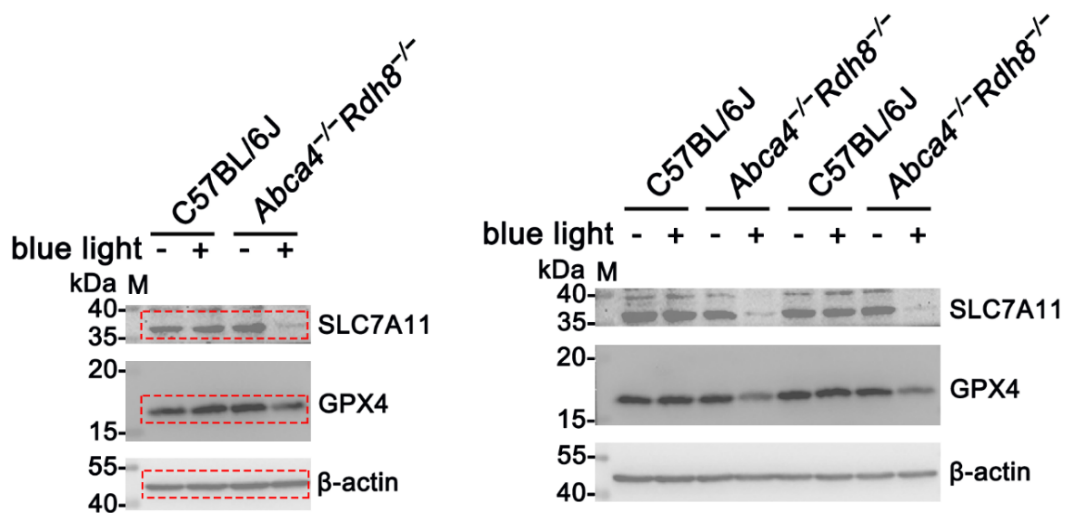


**Figure 6F**


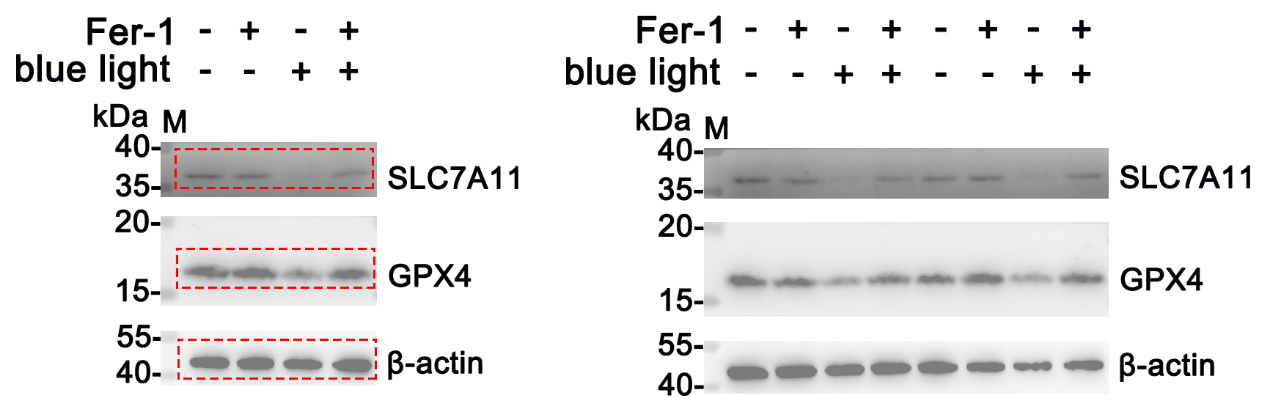


**Supplementary Figure S2.** **Lipid peroxidation is assessed by an image-iT™ lipid peroxidation kit.** **A** Quantification of lipid peroxidation in atRAL-loaded ARPE-19 cells with blue light exposure. **B** Quantification of lipid peroxidation in blue light-exposed atRAL-loaded ARPE-19 cells with GSH treatment. **C** Quantification of lipid peroxidation in blue light-exposed atRAL-loaded ARPE-19 cells with Fer-1 treatment.

**
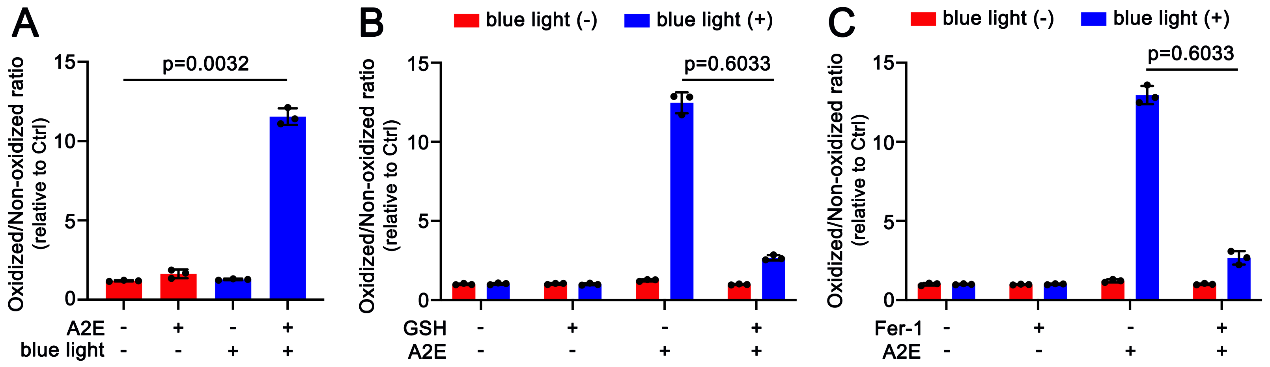
**

**Supplementary Figure S3. Lipid peroxidation is evaluated by acrolein immunofluorescence. A** Quantification of acrolein fluorescence intensity in the RPE/choroid of C57BL/6J and *Abca4^−/−^Rdh8^−/−^* mice with blue light exposure. **B** Quantification of acrolein fluorescence intensity in the RPE/choroid of blue light-exposed *Abca4^−/−^Rdh8^−/−^* mice with Fer-1 treatment.


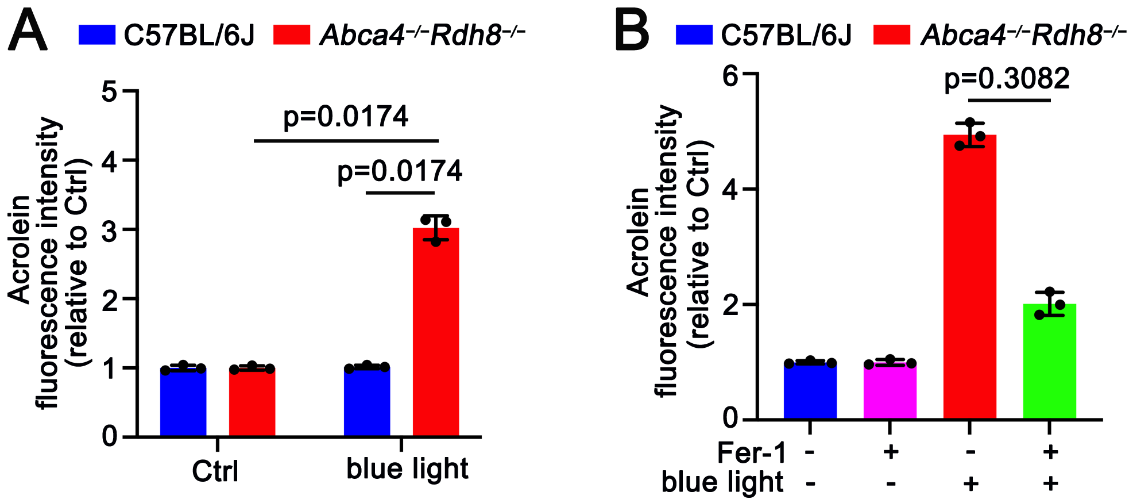


**Supplementary Figure S4. Blue light disrupts iron homeostasis in A2E-loaded ARPE-19 cells.** qRT-PCR analysis of iron homeostasis-related genes in A2E-containing ARPE-19 cells after exposure to blue light. ARPE-19 cells were incubated for 48 h with 25 μM A2E, completely washed with PBS and then placed in fresh DMEM/F12 medium. The cells were illuminated by blue light for 30 min, followed by 24 h of incubation. Control cells were treated with DMSO alone in the absence or presence of blue light.


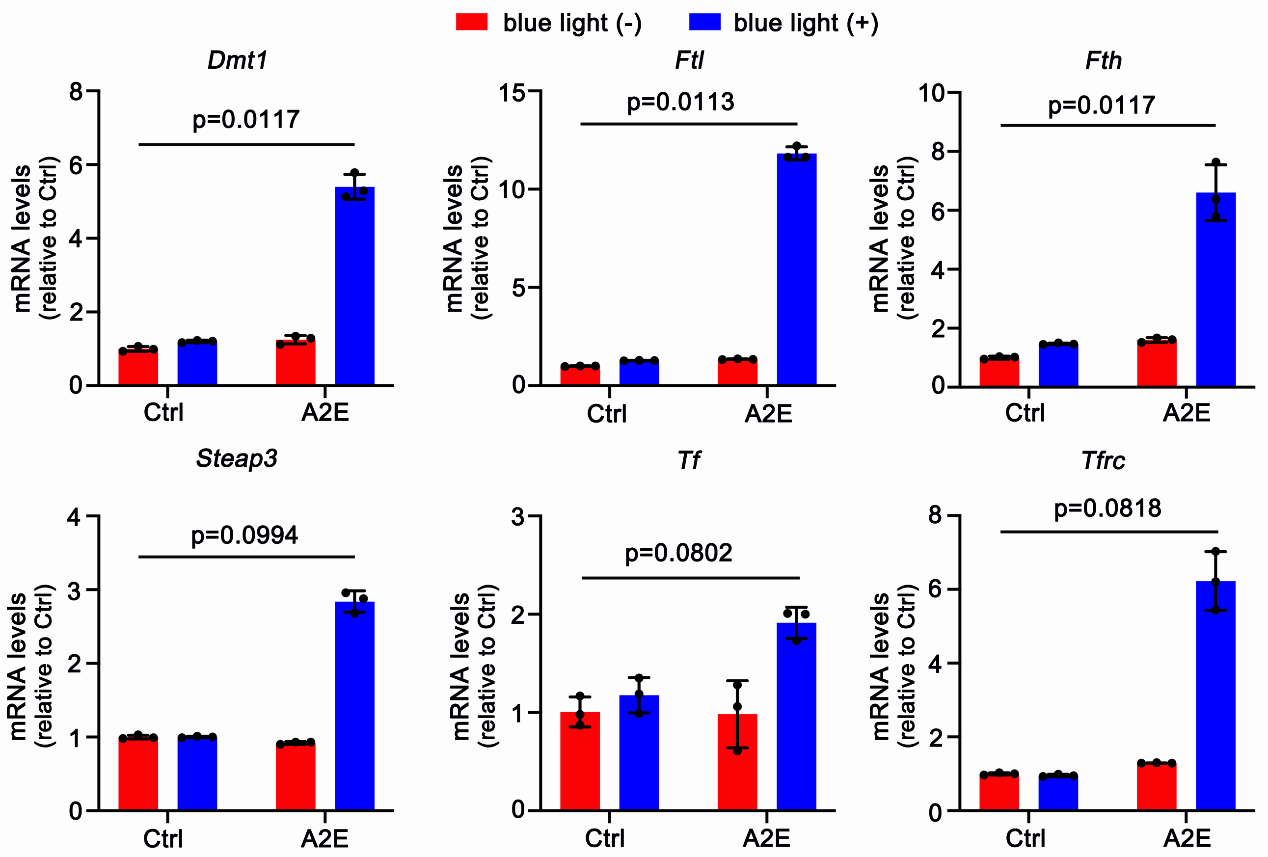

Supplement: Supplementary file 1 — Supplementary Material 1. The online version contains supplementary material. [file 11658_2025_700_MOESM1_ESM.docx]
